# The influence of speleotherapy combined with pulmonary rehabilitation on functional fitness in older adults – preliminary report

**DOI:** 10.1177/1753466620926952

**Published:** 2020-06-10

**Authors:** Sylwia Mętel, Magdalena Kostrzon, Justyna Adamiak, Halina Gattner, Dominika Kościelecka, Angelika Sosulska, Elżbieta Szczygieł, Joanna Golec

**Affiliations:** University of Physical Education in Cracow, al. Jana Pawła II 78, Krakow, 31-571, Poland; ‘Wieliczka’ Salt Mine Health Resort in Wieliczka, Krakow, Poland; University of Physical Education in Cracow, Krakow, Poland; University of Physical Education in Cracow, Krakow, Poland; ‘Wieliczka’ Salt Mine Health Resort in Wieliczka, Krakow, Poland; ‘Wieliczka’ Salt Mine Health Resort in Wieliczka, Krakow, Poland; University of Physical Education in Cracow, Krakow, Poland; University of Physical Education in Cracow, Krakow, Poland

**Keywords:** elderly, subterranean therapy, functional fitness, senior fitness test, healthy ageing

## Abstract

**Objective:**

Our aim was to determine the influence of pulmonary rehabilitation conducted in therapeutic salt mine chambers on the functional fitness of older adults.

**Methods:**

The study included 22 individuals of age >65 years with chronic respiratory conditions. The patients underwent the Fullerton test before and after a 3-week outpatient pulmonary rehabilitation in the “Wieliczka” Salt Mine Health Resort.

**Results:**

After the rehabilitation stay, the results showed statistically significant improvements within five of the six parameters evaluated. In the Arm Curl, the mean number of repetitions within 30 s increased from 14.55 ± 3.63 to 16.68 ± 3.83 and in the Chair Stand from 11.86 ± 2.55 to 14.41 ± 2.95. Beneficial changes were observed in the Back Scratch, but without statistical significance. In Sit and Reach results increased from -2.3 ± 11.11cm to 2.14 ± 9.19 cm. Time for performing the 8-Foot Up and Go decreased from 6.63 ± 1.27 s to 5.8 ± 0.86 s and in 2-Minute Step results increased from 88.27 ± 20.64 to 96.55 ± 16.38 repetitions.

**Conclusion:**

Functional fitness of examined older adults with pulmonary disorders has increased after a rehabilitation and treatment stay in underground salt mine chambers.

*The reviews of this paper are available via the supplemental material section.*

## Introduction

With life expectancy projected to continue rising across all regions of the globe,^1^ the need for preventive and therapeutic measures to alleviate and delay the onset of physical disabilities that accompany ageing become ever more pressing. The World Health Organization (WHO) defines healthy ageing as “the process of developing and maintaining the functional ability that enables wellbeing in older age.”^2^

Pulmonary rehabilitation is a core component of the treatment of patients with chronic respiratory disease and this intervention is beneficial, irrespective of baseline age and levels of disease severity.^3^ Resistance training is considered important for adults to promote healthy aging and also appears to be indicated in individuals with chronic respiratory disease: those who have reduced muscle mass and strength of their peripheral muscles and also suffer from respiratory muscle weakness due to limitations of inspiratory muscle strength and endurance. In pulmonary rehabilitation, an improvement in skeletal muscle function after exercise training lead to gains in exercise capacity despite the absence of changes in lung function.^3,4^

The WHO indicates that the surrounding environment, and particularly pollution, significantly increases the risk of developing an illness.^2^ In order to improve the efficacy of healthy ageing measures, it is recommended to perform physical activities in conditions of good air quality and appropriate climate. Speleotherapy is a special kind of climate therapy which takes advantage of certain conditions specific to caves and historic subterranean salt-mining excavations.^5^

The subterranean atmosphere of the “Wieliczka” Salt Mine is characterized by a high concentration of salt aerosol. The concentration of mineral components is 2.7–8.1 mg/m^3^, causing an osmotic effect which improves the activity of the respiratory epithelium cilia of the upper tracts and bronchi, and also has anti-inflammatory and anti-allergic effects.^6,7^ Other factors that affect the human body in a subterranean environment are: purity of the air, which isolates patients from anthropogenic pollution and allergens; elevated atmospheric pressure, increasing partial pressure of oxygen in the blood; high humidity, preventing excessive drying of the airway epithelium; and favorable ionization.^7–10^

Pulmonary rehabilitation in speleotherapy conditions aims to increase tolerance to physical effort, improve functional fitness including the functioning of the respiratory system, educate the patient with regards to an effective breathing technique and strategy to deal with dyspnea, and also inspire motivation for systematic physical activity.^3^

From the literature review it appears that no research regarding using speleotherapy in pulmonary rehabilitation in older adults has yet been published.

The aim of the study was to determine the efficiency of speleotherapy combined with pulmonary rehabilitation for improvement of functional fitness in older people and to verify if the strength, upper and lower body flexibility, endurance, and dynamic balance in the test group of patients with respiratory disorders are comparable with the standards defined for the Senior Fitness Test (SFT).

## Methods

The initial test group consisted of 26 individuals (21 women and 5 men) referred to a pulmonary rehabilitation program conducted in the “Wieliczka” Salt Mine Health Resort. The test group for the eventual trial included 17 women and 5 men (Table 1).

**Table 1.** General characteristics of the pulmonary patients aged 65 and older.

Variable	*n*%	Mean (SD)
Sex		
Female	17 (77)	
Male	5 (23)	
Age (years)		68.3 (3.01)
Female		68.1 (3.71)
Male		69.8 (2.31)
Weight (kg)		72.2 (8.85)
Height (cm)		161.0 (6.19)
BMI (kg/m^2^)		27.8 (3.75)
Clinical Condition		
Lower respiratory tract disorders	14 (63)	
Asthma	8 (36)	
COPD	5 (23)	
Bronchiectasis	1 (5)	
Upper respiratory tract disorders	8 (36)	
Sinusitis	4 (18)	
Pharyngitis	2 (9)	
Laryngitis	2 (9)	

BMI, body mass index; COPD, chronic obstructive pulmonary disease; SD, standard deviation.

Inclusion criteria were as follows.

1. Chronic upper and lower respiratory tract conditions. The participants were examined by a medical doctor to exclude contraindications for pulmonary rehabilitation and subterranean therapy.^10^
2. Obtaining a minimum of 10 points in the Short Physical Performance Battery test^11^ to assess the risk of disability in older people.
3. Patients aged 65 years and older.
4. Informed, written consent to participate in the project.

The project was carried out on the basis of a study protocol approved by the Bioethical Commission of the Regional Medical Chamber in Krakow (opinion No. 40/KBL/OIL/2018, approval date: March 26, 2018).

All individuals included in the study took the SFT, which includes six consecutive tests:

- 30-second Arm Curl test for upper body strength evaluation;
- 30-second Chair Stand test for lower body strength evaluation;
- Back Scratch test for upper body flexibility evaluation;
- Chair Sit and Reach test for lower body flexibility evaluation;
- 8-Foot Up and Go test for agility/dynamic balance evaluation;
- 2-Minute Step test for endurance evaluation.^12^

SFT, known also as the Fullerton Fitness Test, is a reliable research tool for evaluating the functional fitness of individuals over 60 years old.^13–16^ For this study the assessment was carried out twice: before and after the pulmonary rehabilitation program, according to the recommended procedure, in the same spacious room on the surface and assisted by a group of trained researchers.

## Pulmonary rehabilitation

**Figure 1.**
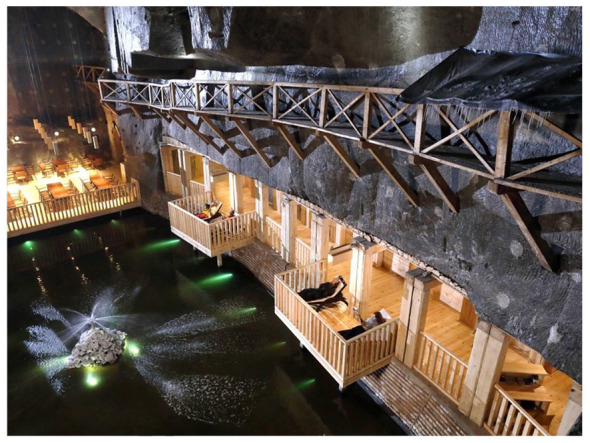
Wessel Lake Treatment Chamber (archive of “Wieliczka” Salt Mine).

The 3-week outpatient pulmonary rehabilitation program included 6-h daily treatment stays in a subterranean salt chamber 5 days a week (Monday–Friday). The underground pulmonary rehabilitation program was performed in a complex of salt chambers in the “Wieliczka” Salt Mine: Wessel Lake Chamber (Figure 1), Eastern Mountains’ Stable, Boczkowski Chamber, and Dragon Chamber (135 m underground).

The pulmonary rehabilitation program included 15 treatment sessions, with 3 sessions (90 min) of supervised, group training run by a physiotherapist. The patients were divided into small groups by a qualified therapeutic team according to the diagnosis given by a qualifying doctor and the patient’s age, comorbidities, and level of physical fitness. The training sessions were implemented according to the following scheme.

- Gait training. Patients went down the shaft and then walked with the assistance of the medical staff along the 500-m long mine corridors to reach the salt chambers complex for the daily exercise program. After the 6-h underground treatment stay, the elderly individuals walked back the same distance to the shaft to exit the mine.
- Strength training of upper and lower limbs for 30 min. Strength training of upper and lower limbs was conducted with the use of dumbbells, elastic bands, gym balls, step platforms, and body weight. In the following weeks, the training was gradually intensified by increasing the load
- Endurance training to music (aerobic exercise) or on a stationary bike for 30 min
- General fitness exercise combined with different breath control strategies for 30 min.

Breathing exercises included pursed lip breathing, breathing control exercises coordinated with physical effort, diaphragmatic breathing, respiratory pattern correction, relaxation, and postural control exercises with the use of neuro-orthopedic activity-dependent plasticity (NAP) therapy.^17^ Each training workout started with a short 5-min warm-up and there was a minimum 20–30-min break between individual types of classes.

An important part of the pulmonary rehabilitation program were health education classes performed additionally two times a week (30 min). The aim of health education was to motivate patients to improve their physical activity, monitor their own health condition and self-esteem, and broaden their knowledge necessary to cope with their health problems. Quantitative comparison of values in two repeated measurements was performed using Student’s *t*-test for paired samples when the variables were normally distributed or Wilcoxon’s tests for paired samples otherwise. Analysis was conducted using the R software package version 3.5.1.^18^

## Results

The study was conducted between March and July 2018. From the initial group of 26 patients, one woman did not complete the full program of pulmonary rehabilitation due to influenza and three women refused to participate in the second examination due to personal reasons. A total of 22 patients were included in the analysis (17 women and 5 men). The age of the participants ranged from 65 to 77 years and the mean age was 68.3 years, (women 68.1, men 68.8). The height of participants ranged from 150 to 174 cm (mean 161.0 cm), body weight ranged from 56 to 96 kg (mean 72.2 kg), and body mass index for the group was between 21 and 35 kg/m^2^ (mean 27.8 kg/m^2^). A normal body weight was registered in 1 person, overweight in 14 individuals and obesity in the remaining 7. Among the conditions taken as an indication for pulmonary rehabilitation combined with speleotherapy, there were chronic diseases of the lower respiratory tract (64%, 14 patients) including: bronchial asthma (8), chronic obstructive pulmonary disease (COPD; 5), and bronchiectasis (1). There were also chronic diseases of the upper respiratory tract (36%, 8 patients) including: sinusitis (4), pharyngitis (2), and laryngitis (2). Among comorbidities, we registered back pain in nine people (40% of patients), lower limb pain in eight people (36% of patients), and upper limb pain in six people (27%). Eight women and one man registered between one and five falls in the last five years.

Analysis of the patients’ functional fitness before and after the pulmonary rehabilitation program was performed, with reference to the standards set for the SFT, regarding the age and sex of the patients.^19^ After trials assessing upper and lower body strength and aerobic endurance, it was found that the majority of individuals obtained results within the norm. For the elasticity of both upper and lower body parts, nearly half of the results (42%, 11 patients) were below the norm in the assessment before the rehabilitation in the underground salt chambers.

After the treatment stay, more than 70% of the elderly patients obtained results in the Sit and Reach test within the norms, however for Back Scratch the results of 8 patients (36% of examined group) were below the standards set by the SFT. In the test evaluating complex coordination, 58% of patients before the pulmonary rehabilitation did not achieve the established standards, but after the treatment stay 64% of the examined seniors fell within the normal range in the agility trial (Table 2).

**Table 2.** The results of the study group’s functional fitness within the American norms before and after the treatment stay in the “Wieliczka” Salt Mine Health Resort. The American norms are constructed in a way that the results within the norm (average) were obtained by 50% of people.

Test	Score	Number and % of patients before the stay	Number and % of patients after the stay
Arm Curl (repeated)	Low	7 (26.92%)	2 (9.09%)
	Medium	18 (69.23%)	15 (68.18%)
	High	1 (3.85%)	5 (22.73%)
Chair Stand (repeated)	Low	9 (34.62%)	0 (0.00%)
	Medium	16 (61.54%)	17 (77.27%)
	High	1 (3.85%)	5 (22.73%)
Back Scratch (cm)	Low	11 (42.31%)	8 (36.36%)
	Medium	9 (34.62%)	6 (27.28%)
	High	6 (23.08%)	8 (36.36%)
Sit and Reach (cm)	Low	11 (42.31%)	2 (9.09%)
	Medium	12 (46.15%)	16 (72.73%)
	High	3 (11.54%)	4 (18.18%)
8-Foot Up and Go (s)	Low	15 (57.69%)	2 (9.09%)
	Medium	9 (34.62%)	14 (63.64%)
	High	2 (7.69%)	6 (27.27%)
2-Minute Step (repeated)	Low	5 (19.23%)	1 (4.55%)
	Medium	18 (69.23%)	15 (68.18%)
	High	3 (11.54%)	6 (27.27%)

In terms of lower body strength after the pulmonary rehabilitation, none of the subjects obtained values below the norm whilst five patients’ performance (23% of patients) was above the norm.

Before the exercise program in subterranean salt chambers, the results for lower body elasticity were found to occupy the low range in 11 seniors (42% of patients), whilst after the treatment stay only two persons were in the low range (9% of patients).

**Figure 2.**
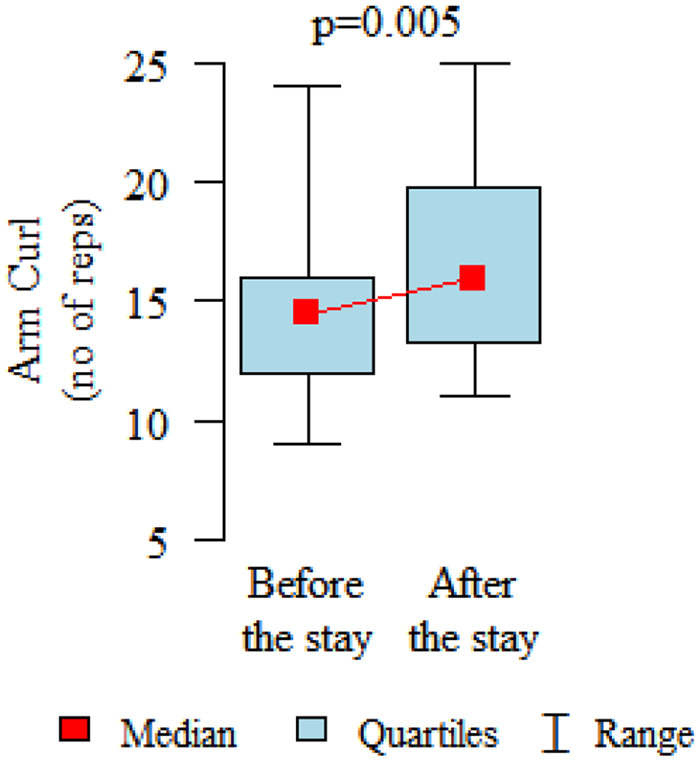
30-second Arm Curl test for upper body strength evaluation.

**Figure 3.**
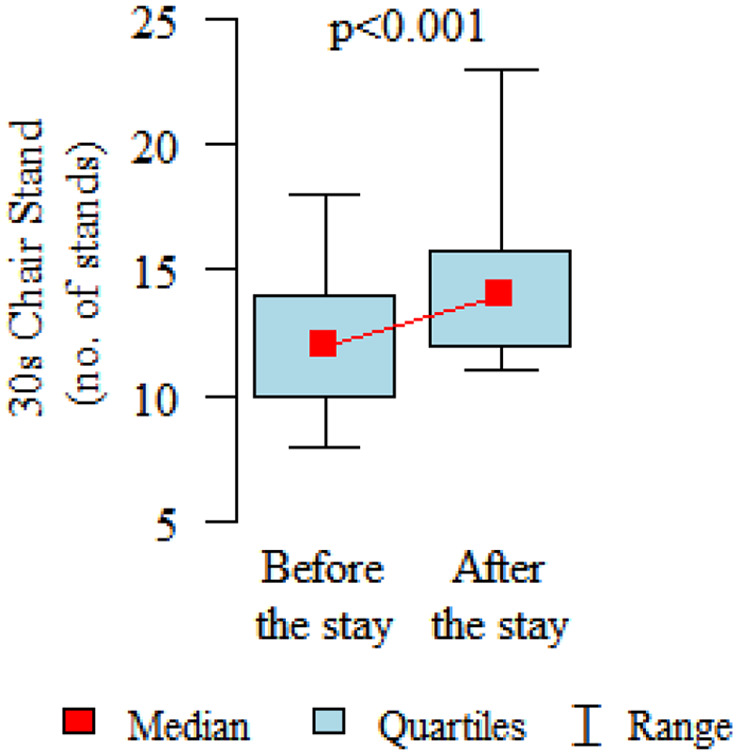
30-second Chair Stand test for lower body strength evaluation.

**Figure 4.**
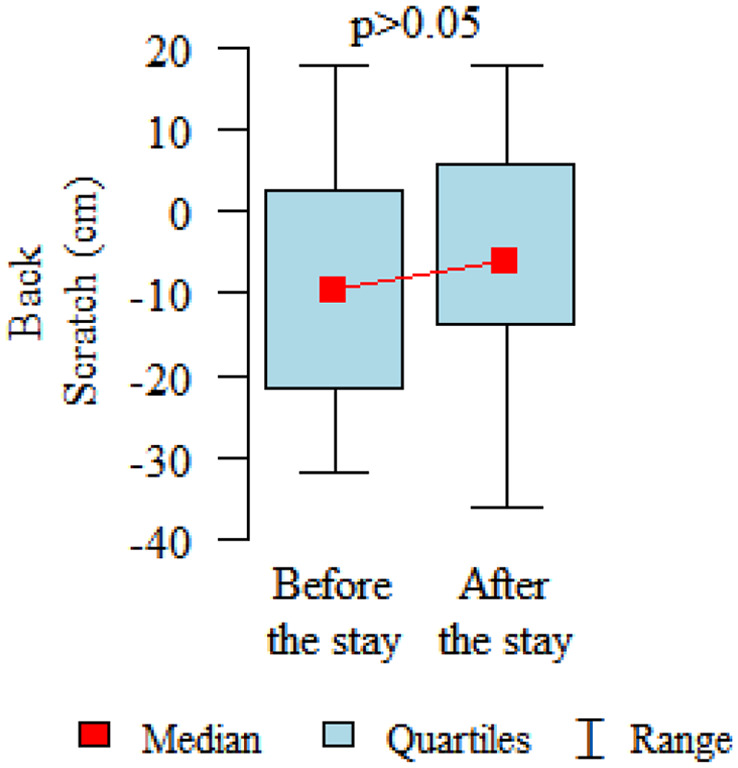
Back Scratch test for upper body flexibility evaluation.

**Figure 5.**
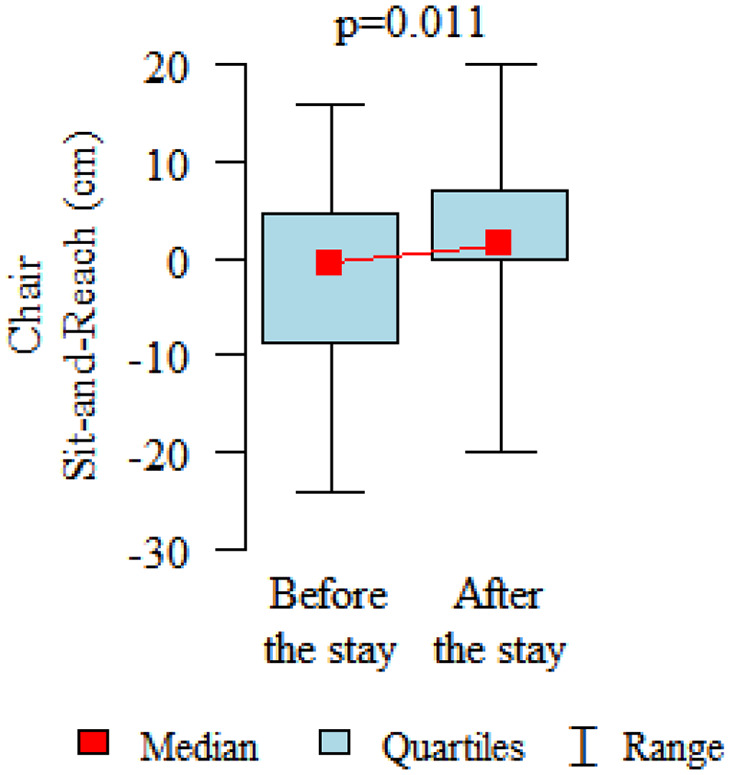
Chair Sit and Reach test for lower body flexibility evaluation.

**Figure 6.**
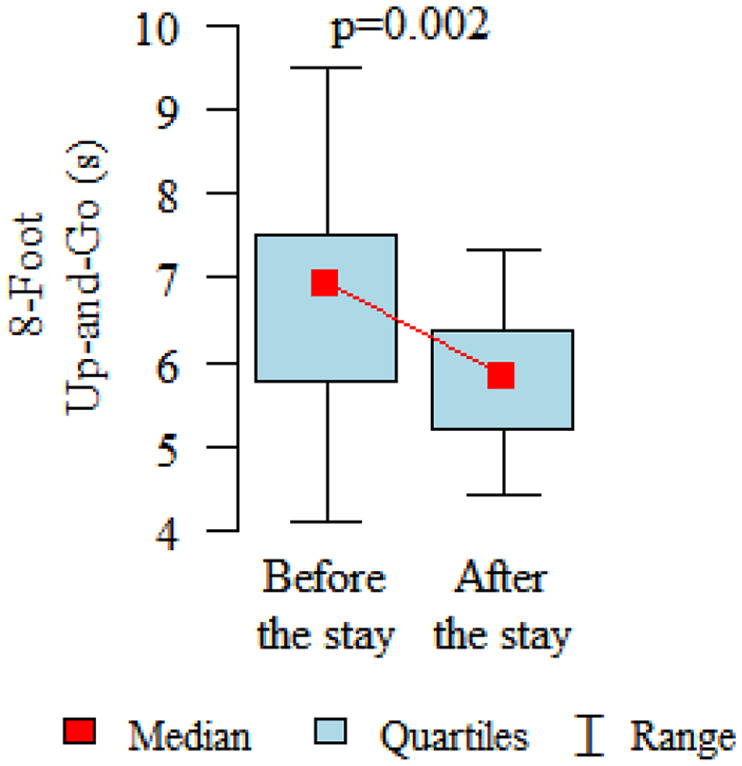
8-Foot Up and Go test for agility/dynamic balance evaluation.

**Figure 7.**
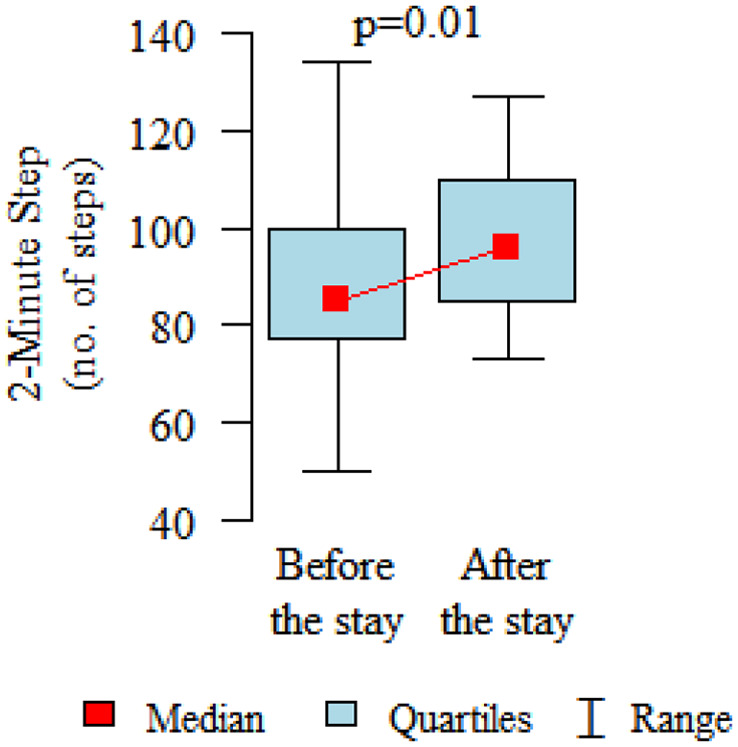
2-Minute Step test for endurance evaluation.

Comparing the pre and post therapy assessments of functional fitness, in five tests (30-Second Chair Stand, 30-Second Arm Curl, 2-Minute Step, Sit and Reach and 8-Foot Up and Go) a significant improvement in performance was observed after the rehabilitation and treatment stay (*p* < 0.05). Only in the elasticity test for the upper body (Back Scratch test) the noted improvement was found to be not statistically significant (Table 3, Figures 2–7). There were no statistically significant differences in functional fitness before the treatment stay within the groups of patients with indications related to the lower and upper respiratory tract. After the stay, the results showed a statistically significant difference only in one out of six parameters evaluated. The mean distance of Chair Sit and Reach test for lower body flexibility evaluation were higher in patients with upper airway indications (Tables 4 and 5).

**Table 3.** The results of the study group’s functional fitness before and after the treatment stay in the “Wieliczka” Salt Mine Health Resort.

Test		Before the stay *n* = 22	After the stay *n* = 22	P
Arm Curl (repeated)	mean ± SD medial quartiles	14.55 ± 3.63 15 12–6.75	16.68 ± 3.83 16 13.25–19.75	0.005 P
Chair Stand (repeated)	mean ± SD medial quartiles	11.86 ± 2.55 12 10–13.75	14.41 ± 2.95 14 12–15.75	< 0.001 P
Back Scratch (cm)	mean ± SD medial quartiles	−7.34 ± 14.26 –7 –20–4.5	−6.07 ± 14.68 –6 –13.75–5.75	0.119 P
Sit and Reach (cm)	mean ± SD medial quartiles	−2.3 ± 11.11 0 –10.5–7.25	2.14 ± 9.19 1.5 0-7	0.011 P
8-Foot Up and Go (s)	mean ± SD medial quartiles	6.63 ± 1.27 6.8 5.58–7.47	5.8 ± 0.86 5.84 5.2–6.39	0.002 P
2-Minute Step (repeated)	mean ± SD medial quartiles	88.27 ± 20.64 85 77.25–100	96.55 ± 16.38 96 84.75–109.75	0.01 P

*p*-value, P-normal distribution of the variations, parametric Student’s *t*-test for dependent measurements (repeated).

**Table 4.** The results of the lower respiratory tract and upper respiratory tract group’s functional fitness before the treatment stay in the “Wieliczka” Salt Mine Health Resort.

Test		Lower respiratory tract group *n* = 14	Upper respiratory tract group *n* = 8	P
Arm Curl (repeated)	mean ± SD medial quartiles	14 ± 2.89 14.5 12.25–16	14,62 ± 4,78 13.5 11.75–16.5	0.682 P
Chair Stand (repeated)	mean ± SD medial quartiles	12.67 ± 2.22 12 11.25–14	10.62 ± 2.62 10 8.75-11.75	0.051 P
Back Scratch (cm)	mean ± SD medial quartiles	−9.31 ± 1.,68 –9.5 –20–1.5	−7.88 ± 13.98 –9 –22–5.5	0.818 P
Sit and Reach (cm)	mean ± SD medial quartiles	1.39 ± 9.78 –0.5 –7.88–4.62	−5.25 ± 11.82 –4.5 –12.25–2	0.392 P
8-Foot Up and Go (s)	mean ± SD medial quartiles	6.77 ± 1.4 6.62 5.58–7.5	6.95 ± 1.33 7.11 6.66–7.56	0,758 P
2-Minute Step (repeated)	mean ± SD medial quartiles	84.44 ± 19.87 81.5 70.25–100.5	95.25 ± 17.91 93.5 82.75–100	0,201 P

*p*-value, P-normal distribution of the variations, parametric Student’s *t-*test for dependent measurements (repeated).

**Table 5.** The results of the lower respiratory tract and upper respiratory tract group’s functional fitness after the treatment stay in the “Wieliczka” Salt Mine Health Resort.

Test		Lower respiratory tract group *n* = 14	Upper respiratory tract group *n* = 8	P
Arm Curl (repeated)	mean ± SD medial quartiles	2.07 ± 3.54 2.5 1–5	2.25 ± 2.71 1.5 1–4	0.756 NP
Chair Stand (repeated)	mean ± SD medial quartiles	2.07 ± 2.84 2 1–3	3.38 ± 1.41 3.5 2.75–4.25	0.242 P
Back Scratch (cm)	mean ± SD medial quartiles	1.43 ± 3.78 0.25 –0.75–2.75	1 ± 3,7 0.5 –1.5–2.5	0.799 P
Sit and Reach (cm)	mean ± SD medial quartiles	1.32 ± 6.13 0 –0.75–4	9.88 ± 6.62 9.5 7-13	0.006 P
8-Foot Up and Go (s)	mean ± SD medial quartiles	0.63 ± 0.98 0.5 0.02–0.73	1.19 ± 1.19 0.92 0.6-1.77	0.162 NP
2-Minute Step (repeated)	mean ± SD medial quartiles	9.71 ± 14,18 10.5 –1.5–21	5.75 ± 13.29 0 –3.25–12	0.526 P

*p*-value, P-normal distribution of the variations, parametric Student’s *t-*test for dependent measurements (repeated); NP, no normal distribution of the variations, non-parametric Wilcoxon’s test for dependent measurements (repeated).

## Discussion

The normal ageing process leads to a deterioration in the function of all tissues and organs of the human body.^20^ Such problems affect the human respiratory system, causing a decline in respiratory efficiency.^21^ There is clearly great potential therapeutic value in identifying methods which are able to both delay this process and to offer preventative benefit.

Pulmonary rehabilitation performed in therapeutic salt chambers offer older adults who suffer from chronic respiratory diseases the possibility to exercise and relax in conditions of microbiological and palynological purity. According to Kostrzon *et al*.^9^ and Olechnowicz-Bobrowska and Wojkowski,^22^ regular monitoring confirms the stability of the underground thermal and humidity conditions and the very low concentrations of micromolecular, respirable dust particulate matter (PM), PM_4_, which is independent of the season and weather above ground and demonstrates the significant air quality in the complex of therapeutic chambers.^8^

Staying in this kind of underground atmosphere free from anthropogenic pollution with a very low concentration of allergens (also in the peak of pollen season), high air quality in terms of bacteriology, and a much lower concentration of micromolecular dust than above ground is very important for the treatment process. The synergistic influence of the aforementioned factors provides a strong therapeutic stimulus with anti-inflammatory, regenerating, and anti-allergic properties.^23^ Moreover, the increased atmospheric pressure and air ionization play a significant role in the treatment process. In the conditions of the “Wieliczka” Salt Mine’s therapeutic chambers, the concentration of light (small) aeroions is 1200–4700 aeroions/cm^3^.^24^ According to Ponikowska and Ferson,^10^ negative air ionization has a good influence on the autonomic nervous system, hormonal system, respiratory tracts, and motor activity of the muscles groups causing, in particular, a reduction in oxygen consumption, an increase in biological activity, and decrease in blood pressure. Taking into consideration all the benefits the atmosphere inside the salt mine chambers can contribute, speleotherapy can be classified as one of the supportive methods for healthy ageing. Exercise training is a major component of pulmonary rehabilitation and therefore exercise performance-related outcomes are consistently used to objectively assess the individual patient’s response to pulmonary rehabilitation and to evaluate the efficacy of the intervention.^3^ When evaluating the functional fitness of senior patients who suffer from pulmonary diseases, it was observed before starting the treatment in the underground resort that the majority of the patients obtained better results than the set American standards with regards to the complex coordination test of the SFT. In three other tests of the pre-treatment trial, that is, upper and lower body strength and endurance, they obtained the results within the established normal range. When flexibility was measured, more than 30% of the participants achieved the required norm for the upper body and nearly half of the group for the lower body. After the treatment stay, more than 70% of individuals reached the normal range in the Sit and Reach test. In the Back Scratch test a significant percentage (42% of the patients before the pulmonary rehabilitation and 36% of the seniors after the treatment program underground) were below the set standards.

In daily life, these physical functional shortcomings can make it difficult to perform activities requiring a raising of the arms or dressing one’s upper body. The authors of this report believe that the presence of pulmonary diseases and the older ages in the group of patients resulted in limitations in body flexibility being present. Shephard^21^ indicates a weakening of the chest muscles progressing with advanced age and mechanical limitations arising from the changing shape of the chest into barrel-chest shape, collapsing of the respiratory tracts during vigorous breathing effort, and changes to the sensory input or central neuronal processing associated with stiffening of the rib cage.

In patients with chronic respiratory diseases there are also postural impairments because respiration and posture have a coupled relationship.^3^ In a controlled study, Lim *et al*.^25^ examined 80 patients with COPD (76 males, 9 females; mean age, 70.6 ± 7.1 years). From their evaluations with the aid of computed tomography, they conclude that the patients with COPD exhibited an increased anterio-posterior diameter of the thoracic cage in comparison with normal controls without any respiratory problems. Szczygieł *et al*.^26^ identified the tendency for the amplitude of rib cage movements to decrease with advancing age. Moreover, these authors point to the expiratory position of the chest and ossification of costal cartilage and shortening of skeletal muscles within the rib cage linked to ageing, which negatively influences both their length-tension relation and their ability to perform mechanical respiratory work.

The analysis of the research results presented in this paper shows that after 15 sessions of a complex pulmonary rehabilitation program in the underground salt chambers, there is an observed significant improvement in performance in five of the six tested parameters of functional fitness in older adults. Only flexibility of the upper body, as evidenced by the Back Scratch test, was not significantly improved after the pulmonary rehabilitation program. A significant improvement within all the parameters of SFT test was found in the research of Carvalho *et al*.^27^ examining the influence of an 8-month multi component physical training program performed twice a week on the functional fitness of women over 65 years old. However, it was further observed that upon discontinuation of the program there was significant deterioration in upper and lower body strength and flexibility after just 3 months, whilst dynamic balance and aerobic endurance were less affected. These authors suggest that emphasis on flexibility training may help to retain functional fitness of older adults.

Mętel *et al*.^28^ used the Sit and Reach test to evaluate the influence of a 6-month sensorimotor training program, performed twice weekly for 50 min on unstable surfaces, on the body flexibility of 37 women aged over 65 years. No significant increase in body flexibility was observed, either immediately upon completing the training program or at a 3-month follow-up assessment.

Douka *et al*.^29^ investigated the influence of traditional Greek dance classes, in twice weekly 75-min sessions over 32 weeks, on a group of 130 people over 60 years old. Employing the SFT, significant positive changes in all functional fitness parameters were observed. As an alternative form of therapeutic intervention that perhaps encourages the continued participation of older adults by its enjoyable nature, and given its potential to increase flexibility in the upper body, it is worth pursuing the inclusion of progressive dance activities or other effective forms of flexibility training in the complex pulmonary rehabilitation of seniors.

The present study suffers for the lack of a control group for comparison, where participants would undertake the same program in regular, over-ground conditions. Nevertheless, the results confirm that promoting healthy ageing should be cross-curricular and take into account functional activities. The SFT test battery enables a consistent trans-global evaluation of older adults’ fitness levels. However, there are no standards agreed for the population of Polish seniors. It would be advisable to additionally formulate them for patients with pulmonary conditions.

## Conclusions

Speleotherapy combined with pulmonary rehabilitation improves the functional fitness of the examined older adults as measured by the SFT, in terms of upper and lower body strength, lower body flexibility, and dynamic balance. Before the treatment stay, the majority of individuals obtained results within the norm established for the SFT in 30-second Arm Curl, 30-second Chair Stand, and 2-Minute Step test. After the pulmonary rehabilitation conducted in subterranean conditions, the results of seniors were not comparable with the standards defined for the Back Scratch test for upper body flexibility evaluation.

As a limitation of this study, it must be noted that there was no control group to go through the same rehabilitation program without the speleological conditions of the salt chamber, which would allow us to identify the relative contributions of both factors to any improvements observed. It is suggested to conduct the same intensive 3-week training program above ground to investigate the impact of climate conditions on the physical performance of seniors.

Achieving an improvement in upper body flexibility in seniors with pulmonary diseases constitutes a therapeutic challenge and may require alternative methods for rehabilitation, for instance with the use of dance or other forms of activity shown to improve this element of functional fitness for older people.

## Supplemental Material

Author_Response – Supplemental material for The influence of speleotherapy combined with pulmonary rehabilitation on functional fitness in older adults – preliminary report Click here for additional data file. Supplemental material, Author_Response for The influence of speleotherapy combined with pulmonary rehabilitation on functional fitness in older adults – preliminary report by Sylwia Mętel, Magdalena Kostrzon, Justyna Adamiak, Halina Gattner, Dominika Kościelecka, Angelika Sosulska, Elżbieta Szczygieł and Joanna Golec in Therapeutic Advances in Respiratory Disease

Reviewer_1_v.1 – Supplemental material for The influence of speleotherapy combined with pulmonary rehabilitation on functional fitness in older adults – preliminary report Click here for additional data file. Supplemental material, Reviewer_1_v.1 for The influence of speleotherapy combined with pulmonary rehabilitation on functional fitness in older adults – preliminary report by Sylwia Mętel, Magdalena Kostrzon, Justyna Adamiak, Halina Gattner, Dominika Kościelecka, Angelika Sosulska, Elżbieta Szczygieł and Joanna Golec in Therapeutic Advances in Respiratory Disease

Reviewer_1_v.2 – Supplemental material for The influence of speleotherapy combined with pulmonary rehabilitation on functional fitness in older adults – preliminary report Click here for additional data file. Supplemental material, Reviewer_1_v.2 for The influence of speleotherapy combined with pulmonary rehabilitation on functional fitness in older adults – preliminary report by Sylwia Mętel, Magdalena Kostrzon, Justyna Adamiak, Halina Gattner, Dominika Kościelecka, Angelika Sosulska, Elżbieta Szczygieł and Joanna Golec in Therapeutic Advances in Respiratory Disease

Reviewer_2_v.1 – Supplemental material for The influence of speleotherapy combined with pulmonary rehabilitation on functional fitness in older adults – preliminary report Click here for additional data file. Supplemental material, Reviewer_2_v.1 for The influence of speleotherapy combined with pulmonary rehabilitation on functional fitness in older adults – preliminary report by Sylwia Mętel, Magdalena Kostrzon, Justyna Adamiak, Halina Gattner, Dominika Kościelecka, Angelika Sosulska, Elżbieta Szczygieł and Joanna Golec in Therapeutic Advances in Respiratory Disease

Reviewer_2_v.2 – Supplemental material for The influence of speleotherapy combined with pulmonary rehabilitation on functional fitness in older adults – preliminary report Click here for additional data file. Supplemental material, Reviewer_2_v.2 for The influence of speleotherapy combined with pulmonary rehabilitation on functional fitness in older adults – preliminary report by Sylwia Mętel, Magdalena Kostrzon, Justyna Adamiak, Halina Gattner, Dominika Kościelecka, Angelika Sosulska, Elżbieta Szczygieł and Joanna Golec in Therapeutic Advances in Respiratory Disease
