# Supplementary material for: The influence of speleotherapy combined with pulmonary rehabilitation on functional fitness in older adults – preliminary report
Source: Ther Adv Respir Dis. 2020 Jun 10;14:1753466620926952. doi: 10.1177/1753466620926952 (PMC7288829; doi:10.1177/1753466620926952)
Supplement: Reviewer_1_v.1 – Supplemental material for The influence of speleotherapy combined with pulmonary rehabilitation on functional fitness in older adults – preliminary report [file Reviewer_1_v.1.pdf]

Reviewer 1 v.1

Comments to the Author

The topic is relevant as an add on therapy of pulmonary rehabilitation.

More imformation is needed about lung function. It can improve the strength of the publication if it contains lung mechanics, peripheral and respiratory muscle strength if it is available.

I suggest some classical rehabilitation study as a reference, like Casaburi et al., Spruit et al.

Eglish language correction is needed.

With kind regards,
