# Supplementary material for: The influence of speleotherapy combined with pulmonary rehabilitation on functional fitness in older adults – preliminary report
Source: Ther Adv Respir Dis. 2020 Jun 10;14:1753466620926952. doi: 10.1177/1753466620926952 (PMC7288829; doi:10.1177/1753466620926952)
Supplement: Reviewer_1_v.2 – Supplemental material for The influence of speleotherapy combined with pulmonary rehabilitation on functional fitness in older adults – preliminary report [file Reviewer_1_v.2.pdf]

Reviewer 1 v.2

Comments to the Author

Dear Author,

The quality of the manuscript improved with the suggestions. The weakness of the study is the low number of participants.

With kind regards,
