# Supplementary material for: The influence of speleotherapy combined with pulmonary rehabilitation on functional fitness in older adults – preliminary report
Source: Ther Adv Respir Dis. 2020 Jun 10;14:1753466620926952. doi: 10.1177/1753466620926952 (PMC7288829; doi:10.1177/1753466620926952)
Supplement: Reviewer_2_v.1 – Supplemental material for The influence of speleotherapy combined with pulmonary rehabilitation on functional fitness in older adults – preliminary report [file Reviewer_2_v.1.pdf]

Reviewer 2 v.2

Comments to the Author

Dear authors,

Thank you for the opportunity to review your manuscript. In the present study, the authors compared physiological responses after performing a pulmonary rehabilitation programme in the salt mine chamber in patients with chronic respiratory diseases. Although the topic is useful for daily clinical practice, the study has its methodological limitations. As the authors mentioned in their manuscript, there is a lack of a control group. Moreover, the tested group is not homogenous. Therefore I have some considerations that could influence the article quality:

1. It is necessary to change your conclusion in the abstract part of your manuscript. You are not able to conclude that your study confirmed the health benefits of the underground air because you didn’t have any control group in your study (the same outpatient pulmonary rehabilitation programme in a gym).

2. The introduction part is quite long and not all paragraphs are associated with the topic of this manuscript. Some of them are general.

3. Please specify your group of patients more in detail. Please add the numbers of patients according to their diagnosis and add also the severity of their disease (e.g. stage, lung functions) and the length of their disease.

4. Why did you perform your study in 26 patients? Did you use any sample size calculation?

5. Your group of patients is not homogenous (different diseases with different pathophysiology) what can influence your results. From my point of view, it should be better to compare the effect of pulmonary rehabilitation treatment on the functional status between patients with different diseases – e.g. COPD and asthma, or COPD and bronchiectasis, etc. But it is necessary to have enough patients in each group.

6. Please specify the pulmonary rehabilitation programme in more detail. The pulmonary rehabilitation programme is written in general, it is not possible to use your programme in another study.

7. Please add detailed information about functional fitness in your patients after pulmonary rehabilitation in table 2 in Results.

8 Please add detailed information about functional fitness in your patients before and after rehabilitation programme in association with different diagnoses in Results.

9. Please add statistical significance to your figures. In figure 2 correct the title – add number 30 (30-second chair stand test….).
