# Supplementary material for: The influence of speleotherapy combined with pulmonary rehabilitation on functional fitness in older adults – preliminary report
Source: Ther Adv Respir Dis. 2020 Jun 10;14:1753466620926952. doi: 10.1177/1753466620926952 (PMC7288829; doi:10.1177/1753466620926952)
Supplement: Reviewer_2_v.2 – Supplemental material for The influence of speleotherapy combined with pulmonary rehabilitation on functional fitness in older adults – preliminary report [file Reviewer_2_v.2.pdf]

Reviewer 2 v.2

Comments to the Author

Dear Authors,

Thank you very much for your revision of your manuscript. Your manuscript is written better now. Although the topic is interesting and important for clinical practice, I think that it is still necessary to improve the methodology of your study. Because it is not easy to interpret your results now. If it is possible, use the same training program in a randomized controlled study, calculate sample size, assess homogenous group with detailed specification of patient's disease (different results should be found for patients with the milder disease compared to more severe disease, also the length of the disease should influence the results, etc.). There are not enough studies focused on the combination of speleotherapy with pulmonary rehabilitation, your work is very important, but confirmation of the effect of this treatment combination needs high-quality studies.
