# Supplementary material for: The influence of speleotherapy combined with pulmonary rehabilitation on functional fitness in older adults – preliminary report
Source: Ther Adv Respir Dis. 2020 Jun 10;14:1753466620926952. doi: 10.1177/1753466620926952 (PMC7288829; doi:10.1177/1753466620926952)
Supplement: Author_Response – Supplemental material for The influence of speleotherapy combined with pulmonary rehabilitation on functional fitness in older adults – preliminary report [file Author_Response.pdf]

Author Response

The authors would like to thank both reviewers for all comments, suggestions and corrections, which have been considered.

Reviewer 1

The topic is relevant as an add on therapy of pulmonary rehabilitation. More information is needed about lung function. It can improve the strength of the publication if it contains lung mechanics, peripheral and respiratory muscle strength if it is available.

I suggest some classical rehabilitation study as a reference, like Casaburi et al., Spruit et al.

English language correction is needed.

Authors:

Additional information about lung and muscle function in patients with chronic respiratory diseases has been included in the manuscript with a reference to suggested authors.

The English language of the manuscript has been improved as suggested by the reviewer. Native English speaker has done proofreading. If the correction is not satisfactory, please clarify what needs to be improved.

Reviewer: 2

Thank you for the opportunity to review your manuscript. In the present study, the authors compared physiological responses after performing a pulmonary rehabilitation program in the salt mine chamber in patients with chronic respiratory diseases. Although the topic is useful for daily clinical practice, the study has its methodological limitations. As the authors mentioned in their manuscript, there is a lack of a control group. Moreover, the tested group is not homogenous. Therefore I have some considerations that could influence the article quality:

1. It is necessary to change your conclusion in the abstract part of your manuscript. You are not able to conclude that your study confirmed the health benefits of the underground air because you didn't have any control group in your study (the same outpatient pulmonary rehabilitation program in a gym).

Authors:

Conclusion in the abstract part of the manuscript has been changed according to the Reviewer's relevant remarks. *Conclusion: Functional fitness of examined, older adults with pulmonary disorders has increased after a rehabilitation and treatment stay in underground, salt mine chambers.*

2. The introduction part is quite long and not all paragraphs are associated with the topic of this manuscript. Some of them are general.

Authors:

Some general information has been excluded from the introduction part of the manuscript.

3. Please specify your group of patients more in detail. Please add the numbers of patients according to their diagnosis and add also the severity of their disease (e.g. stage, lung functions) and the length of their disease.

Authors:

The examined group of patients has been specified more in detail: the numbers of patients according to their diagnosis have been added in the Results and in Table 1. Unfortunately, we are not able to add the severity of the diseases and the length of our patients' diseases.

4. Why did you perform your study in 26 patients? Did you use any sample size calculation?

Authors:

Initially, there were 26 participants recruited through volunteer sampling. During the study period 1 of them did not finished the whole program of the treatment stay due to medical condition and the other 3 refused to participate in the second examination in the research. We did not use the sample size calculation.

5. Your group of patients is not homogenous (different diseases with different pathophysiology) what can influence your results. From my point of view, it should be better to compare the effect of pulmonary rehabilitation treatment on the functional status between patients with different diseases – e.g. COPD and asthma, or COPD and bronchiectasis, etc. But it is necessary to have enough patients in each group.

Authors:

Thank you for your feedback. We appreciate your suggestion and take it into consideration with future research project.

6. Please specify the pulmonary rehabilitation program in more detail. The pulmonary rehabilitation program is written in general, it is not possible to use your program in another study.

Authors:

The pulmonary program has been described in more detail as suggested by the reviewer.

7. Please add detailed information about functional fitness in your patients after pulmonary rehabilitation in table 2 in Results.

Authors

Information about functional fitness of the patients after pulmonary rehabilitation has been implemented in table 2 in Results.

8. Please add detailed information about functional fitness in your patients before and after rehabilitation programme in association with different diagnoses in Results.

Authors

There were no statistically significant differences in functional fitness before the treatment stay within the groups of patients with diagnosis related to the lower and upper respiratory tract. After the treatment stay, the results showed statistically significant difference only in one out of six parameters evaluated. The mean distance of Chair Sit and Reach test for lower body flexibility evaluation were higher in patients with upper airway diagnosis.

9. Please add statistical significance to your figures. In figure 2 correct the title – add number 30 (30-second chair stand test….).

Authors

Statistical significance has been added to the Figures, in Figure 2 title has been corrected.
